# A method for rapid testing of social learning in vampire bats

**DOI:** 10.1098/rsos.172483

**Published:** 2018-06-27

**Authors:** Julia K. Vrtilek, Gerald G. Carter, Krista J. Patriquin, Rachel A. Page, John M. Ratcliffe

**Affiliations:** 1 Department of Biology, Eidgenössische Technische Hochschule Zürich, Zürich, Switzerland; 2 Department of Collective Behaviour, Max Planck Institute for Ornithology, Radolfzell, Germany; 3 Department of Biology, University of Konstanz, Konstanz, Germany; 4 Department of Biology, University of Toronto Mississauga, Mississauga, Canada; 5 Smithsonian Tropical Research Institute, Apartado 0843-03092, Balboa, Ancón, Republic of Panama

**Keywords:** maze, social learning, spatial memory, vampire bats

## Abstract

Designing experiments on social learning using an untested behaviour or species requires baseline knowledge of how the animals will perform. We conducted a pilot study of a procedure for rapidly testing social learning in the highly social common vampire bat (*Desmodus rotundus*) using a simple maze. To create demonstrators, we allowed captive bats to learn to exit a three-dimensional maze, which reunited them with their colony as a reward. We then filmed naive bats in the same maze, comparing their ability to exit the maze before, during and after the addition of a trained demonstrator. The presence of a demonstrator increased the exit rates of naive bats, presumably by attracting the attention of the naive bats to the maze exit. Four of the five naive bats that exited in the presence of a demonstrator retained the ability to exit without the demonstrator. No naive bat exited during trials without a potential demonstrator present. This experimental procedure appears to be a promising approach for efficient tests of social learning in vampire bats because maze difficulty can be manipulated to adjust learning rates and each trial requires only 15 min.

## 1. Introduction

Many animals gather information from observing the behaviours of others [1]. Most studies of such social learning have involved experiments on the social effects of foraging or mate choice [1,2]. To generalize further about social learning, it is useful to test a variety of other adaptive behaviours that can be quickly assessed and easily manipulated, such as the learning of escape or exit routes [3,4]. In order to study social learning using novel tasks in non-model organisms, pilot tests are necessary to determine what factors can be reliably manipulated, how quickly the animals will learn and which experimental designs are most promising.

We assessed the feasibility of a procedure for rapid testing of social learning in the common vampire bat (*Desmodus rotundus*). The common vampire bat is a highly social species in which both kin and non-kin form long-term cooperative relationships that involve allogrooming and regurgitated food sharing [5–8]. There are several ways that vampire bats might use social information for spatial learning. On a large scale, social cues may help bats learn about and locate sites with reliable aggregations of potential hosts (prey), such as livestock or sea lions [9,10]. On a smaller scale, other bats could influence whether and how to approach novel objects or prey [11].

In this study, we tested whether the presence of trained demonstrators would increase the ability of naive vampire bats to exit a three-dimensional maze. Most social learning experiments have used a food reward for training, but this requires limiting food to create food-motivated subjects and constrains the number of trials that can be conducted in a day. We instead used a social reward in training, as vampire bats are highly social and motivated to re-join their social group [5]. Successful exits from the maze allowed bats to return to their groupmates in the home cage. After training demonstrators, we tested triads of naive bats in the same maze and compared their naive exit rates before, during and after the presence of a trained demonstrator.

We considered five hypotheses. First, if the naive bats did not learn exit routes (*no learning*), we expected no increase in exit performance across trials. Bats that did exit would not be faster or more likely to exit again compared with naive bats that failed to exit. Second, if the naive bats learned the exit route on their own through trial and error (*asocial learning*), we expected that exit rates should improve, but that the change in naive exit rates between the first and second trials in the test phase (i.e. before and after the addition of a demonstrator) should not be greater than the same change between the first and second sessions of solitary naive bats in the training phase. Third, if naive bats followed the demonstrator out of the maze but did not learn (*following without learning*), the addition of demonstrators should increase naive exit rates and we should see the naive bat follow and exit immediately after the demonstrator, but the naive exit rate should then decrease when the demonstrator is removed. Fourth, the naive bats might follow the demonstrator and then retain the ability to exit when the demonstrator is removed (*social learning from following*). Fifth and finally, the naive bats might not directly follow the demonstrator out of the maze, but the demonstrator might still increase the exit rate of naive bats (*social learning without following*). In this case, the addition of a demonstrator should increase naive bat exits, the newly exiting bats should retain the ability to exit when the demonstrators are removed, and the change in exit rates between the first and second test trials (with demonstrator added) should be greater than the same change between the first and second training sessions (no demonstrator added).

## 2. Material and methods

### 2.1. Experiment overview

**Figure 1.**
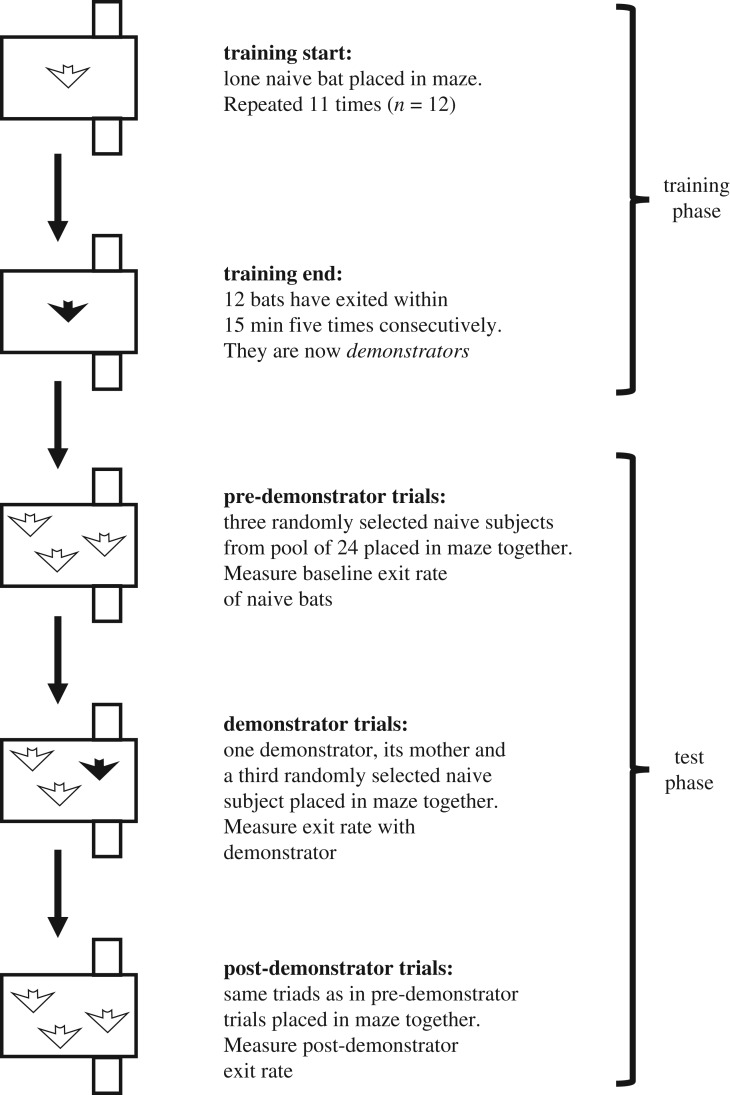
Schematic of experimental design. Black bats are trained demonstrators and white bats are naive subjects (except in post-demonstrator trials, at which point the subjects may have learned the exit).

In the training phase, we individually trained 12 bats to exit the three-dimensional maze back into their home cage. These bats then became *demonstrators*. In the testing phase, we used the same maze to measure the baseline exit rate across 24 bats tested as eight triads, each with three naive subjects (pre-demonstrator test trials). Next, we measured naive exit rates across 24 naive bats in 12 triads, each with two naive subjects and one trained demonstrator (demonstrator test trials). Finally, we again measured the exit rates across the initial eight triads of subjects (post-demonstrator test trials, figure 1).

### 2.2. Animal subjects

The experiment involved 36 common vampire bats (*D. rotundus*). All subjects were either captured in Panama or born in captivity, individually marked with unique metal forearm bands, and housed as a group in a 1.7 × 2.1 × 2.3 m outdoor flight cage in Gamboa, Panama. Bats were fed twice every night with silo-type bird water spouts filled with bovine blood defibrinated with 11 g sodium citrate and 4 g citric acid per 3.8 l of blood. Twelve captive-born bats (ages 4–13 months) served as demonstrators. Twenty-four older wild-caught female bats served as naive subjects that could potentially learn from the demonstrators. We used younger bats as demonstrators because it allowed us to pair each demonstrator with its mother and one unrelated female during demonstrator trials, allowing the possibility of testing kin-biased social learning. We have also observed that younger bats are more exploratory [11], and thus we suspected they were more likely to solve the maze on their own.

### 2.3. Test maze design

**Figure 2.**
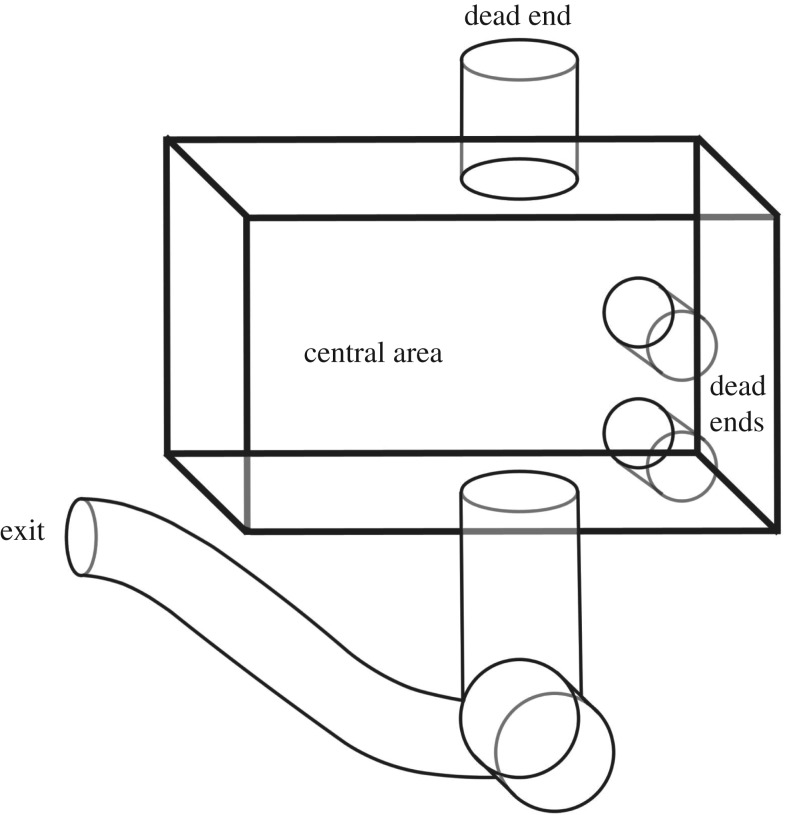
Schematic of maze. Exit hatch can be pushed outwards into the main colony cage, but not inwards to enter the maze.

The test maze consisted of a central wooden box frame with four mesh sides, two transparent plastic sides for visibility when recording video, three mesh exit arms that were dead ends, and one mesh exit arm leading to a longer plastic tube that ended in a metal door-flap (figure 2). The exit flap was a one-way door: bats that pushed open the flap emerged into the room containing the rest of the colony, but the flap could not be entered from the other side by other colony members. To start the maze, bats were placed in the central box area. The box was not large enough to fly in, but the bats could walk, climb and jump, all of which are natural behaviours for vampire bats.

### 2.4. Training sessions

We allowed the 12 demonstrator bats to learn to exit the maze over 11 sessions. In the first training session, we placed each bat individually inside the maze for at least 2 h, and if the bat had not exited by the end of the session, we removed it from the maze. In the second training session, we left each bat in the maze for at least the amount of time it had taken that bat to exit previously, or for at least 2 h if they had never successfully exited. In the next four training sessions, bats that had previously taken more than 10 min were left in the maze for at least the amount of time it had taken the bat to exit previously, and bats that had previously exited in under 10 min were not re-tested. In the final five sessions, all bats were tested individually in consecutive trials. Bats were considered ready to serve as demonstrators when they had exited in under 15 min on five consecutive occasions.

### 2.5. Test trials

We refer to bats that have never solved the maze as *naive*. In pre-demonstrator trials, we randomly assigned 24 naive bats to eight groups of three subjects, and each unique triad of naive bats was put into the maze and filmed for 15 min. In demonstrator trials, each demonstrator bat was paired with its mother and another random naive subject to create a new unique triad that was put into the maze and filmed for 15 min. In post-demonstrator trials, we repeated the procedure for the pre-demonstrator trial to determine whether previously exiting bats retained the ability to exit without a demonstrator. After all trials, non-exiting bats were captured and returned to their home cage.

### 2.6. Statistical analysis

The ‘naive exit rate’ for each trial type is the number of naive subjects that exited within the 15 min trial divided by the number of possible naive exits. We defined ‘exit time’ as the latency until the subject exited within the 15 min trial. For testing changes in exit times across test trial types, subjects that never exited were assigned an exit time of 15 min. To assess whether learning took place during the training phase, we plotted the exit times for each subject during each training session. To test whether naive exit rates increased with the addition of a demonstrator, we fitted a generalized linear mixed model (GLMM; binomial distribution, logit link function, lme4 package in R) with the presence or absence of a demonstrator as a fixed effect, subject as a random effect and failure or exit (0 or 1) as the response. As a *post hoc* test, we used the mid-p McNemar test [12] to determine whether the exit rates increased from the pre-demonstrator to the demonstrator trial, and whether they changed again from the demonstrator to the post-demonstrator trial.

One might expect the naive exit rate to decrease for the demonstrator trial simply because bats that were successful in the pre-demonstrator trial would be removed from the pool of possible learners. Alternatively, one might expect the naive exit rate to increase for the demonstrator trial because the remaining naive bats will have had more time to learn about the maze: a naive bat will have spent 15 min in the maze at the end of the first attempt (pre-demonstrator trial) and 30 min at the end of the second attempt (demonstrator trial). To account for these possible effects, we used Fisher's exact test to compare the counts of naive bats in test trials versus the counts of naive bats in training sessions that successfully exited in 15 min on their first attempt, second attempt or neither. In the test trials, a demonstrator was added for the second attempt; the training sessions can serve as a control because neither the first nor second attempt included a demonstrator.

To test whether exiting bats retained knowledge of the exit, we also used Fisher's exact test to compare the number of naive and experienced bats that exited in the post-demonstrator trials without a potential demonstrator. To compare the effect of demonstrators on exit times for naive bats, we used non-parametric bootstrapping (5000 iterations, boot package, BCa method [13]) to calculate the 95% confidence intervals around the mean exit time for pre-demonstrator, demonstrator and post-demonstrator test trials. We did not attempt to estimate odds ratios when contingency tables contained zeros, leading to zero denominators in the odds ratio estimates. Data and R code for all tests are available in the electronic supplementary material.

## 3. Results

### 3.1. Demonstrator training sessions

**Figure 3.**
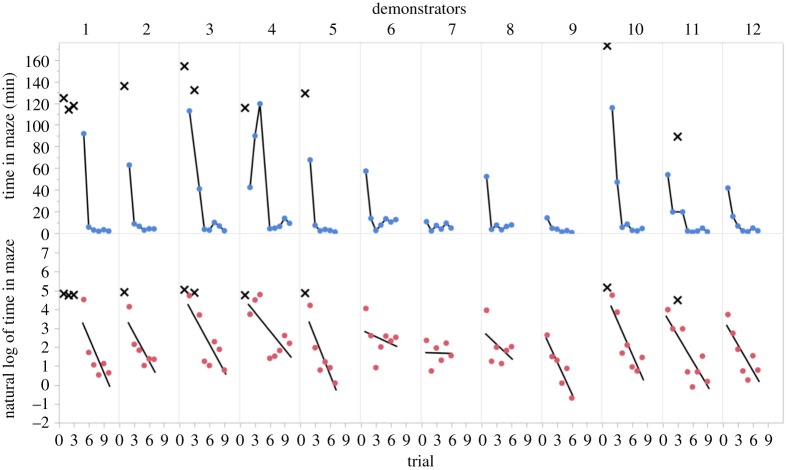
Improvement in exit times during training sessions. Time in the maze during each training session until exit (point) or until the end of the session with no exit (cross symbols) are plotted in minutes (top panel) or log-transformed minutes with a line of best fit (bottom panel) for each of 12 demonstrator bats.

During the first training session, two of 12 naive bats exited within 15 min and four additional naive bats exited after more than 15 min. During the second training session, none of the six remaining naive bats exited within the first 15 min. The mean time that naive bats spent in the maze before exiting was 149 min (95% CI: 86 to 285 min, median = 154 min, range = 11–569 min). By the end of the training phase, all bats had exited in under 15 min on five consecutive occasions and could serve as demonstrators (figure 3).

### 3.2. Test trials

During demonstrator trials, exit times of the demonstrators were similar to their final training session (mean difference in exit time = 2 min faster, 95% CI: 7 min faster to 2 min slower); six demonstrators exited slower, five exited faster, and one did not completely exit within 15 min.

The proportion of naive bats that exited the maze increased with the presence of a demonstrator (GLMM, odds ratio = 11.4, *p* = 0.0315; McNemar test: $\chi^{2}=4.17$, d.f. = 1, *p* = 0.04). During pre-demonstrator trials, none of the 24 naive subjects exited. During demonstrator trials, five of the 23 naive subjects exited. To be conservative, we excluded one of the 24 naive subjects from our analysis because it exited the maze after the pre-demonstrator trial while being captured and removed from the maze by an experimenter.

In post-demonstrator trials, the absence of the demonstrator did not significantly change the overall exit rate (McNemar test: $\chi^{2}<0.1$, d.f. = 1, *p* > 0.9). Four of the five bats that had previously exited with demonstrators exited again. Of the remaining 18 naive subjects, one exited for the first time in the post-demonstrator trial. This was the only naive exit to occur in the 24 test trials without demonstrators; however, this bat may also have learned socially, because it exited after an experienced bat that played the same role as a demonstrator (table 1). None of the 17 naive bats without previous exit experience or a possible demonstrator exited during a test trial (0/17 naive bats versus 5/6 experienced bats, Fisher's exact test, 95% CI of odds ratio = 0 to 0.16, *p* < 0.001).

**Table 1.** Latency to exit (minutes) for subjects that learned to exit. Seventeen other bats did not learn to exit.

bat	pre-demonstrator	demonstrator	post-demonstrator
D	no exit (>15)	5.0	no exit (>15)
DD	no exit (>15)	5.0	4.5
DS	no exit (>15)	8.6	3.6
R^a^	no exit (>15)	9.1	3.0
S	no exit (>15)	9.7	5.7
SD	no exit (>15)	6.2	5.7
SS	no exit (>15)	no exit (>15)	7.1^b^

^a^Bat R exited after the 15 min pre-demonstrator trial and was removed from the analysis to be conservative.

^b^Subject could have been influenced by another subject previously exiting in the same trial.

As expected if the increase in the naive exit rate was not due to increased time spent in the maze, the change in the probability of naive exits from the first to the second time in the maze was greater for test trials than for training sessions (Fisher's exact test, *p* = 0.0429). In test trials, the success rate increased from 0/24 naive bats exiting during their first attempt to 5/23 naive bats exiting during their second attempt when a demonstrator was present. By contrast, none of the six bats remaining naive after the first training session succeeded during the first 15 min of their second attempt, despite having spent more than 2 h in the maze and despite showing greater exploration rates [11].

The mean naive exit time in the presence of a demonstrator (mean = 13.2 min, 95% CI = 11.3 to 14.4 min) was faster than in the pre-demonstrator trial (mean = 15 min, no exits) but not significantly different from the post-demonstrator trial (mean = 14.6 min, 95% CI = 12.8 to 15.0 min, table 1).

The individual behaviours leading to the naive exits in the test trials give insight into the mechanism by which those exits occurred. In the first naive exit of the demonstrator test trials, the demonstrator entered the exit arm and exited the maze via the exit flap, then the naive subject exited 1.7 min later. In the second and third naive exits, a naive subject entered a non-exit arm, the demonstrator joined it 9 s later, then the demonstrator left the naive subject behind and entered the exit arm. A naive subject joined the demonstrator 9 s later, and the second naive subject joined them both 1.4 min later. Both naive subjects exited together via the exit flap, and the demonstrator exited 1.3 min later. In the fourth naive exit, the demonstrator entered the exit arm and moved towards the exit; a naive subject joined 27 s later, left the exit arm, and then returned, re-joining the demonstrator. The naive subject then exited via the exit flap and the demonstrator exited 4.7 min later. In the fifth naive exit, the demonstrator bat entered the exit arm, then both naive subjects entered the exit arm. The demonstrator then exited, and one of the two naive subjects in the arm exited 7.7 min later.

## 4. Discussion

Our results show that mazes have potential for testing spatial and social learning in vampire bats. Although navigating a maze and pushing a flap door is a novel task, the act of crawling through a structured space is not entirely artificial, because wild vampire bats walk and hop through very tight passages in caves where flight is not possible (G. Carter 2007, 2017, personal observations in Mexico and Panama). Of all bats, this species is the most adept at terrestrial locomotion [14,15] and can easily move about in a maze or similar apparatuses designed for rodents. We caution that such mazes are unlikely to work as well for other bat species that are not as proficient at walking.

Our goal was to design a maze difficult enough that bats could not immediately discover the exit route, but simple enough that they could remember the exit route after a single exit. This was successful in that bats learned to exit the maze relatively quickly (within a few hours) and naive bats were unable to exit as quickly as trained bats. An earlier version of the maze with a simpler exit route leading straight to the home cage proved to be too easy, as naive bats exited very quickly, potentially aided by the sound or smell of the bats in the home cage. We therefore adjusted the maze so that the exit route initially led away from the home cage and the door could not be seen from the starting point. If bats can detect the exit from the entrance of the maze, there is nothing to learn, but if they cannot, they are unable to socially learn the second task of opening the door unless directly behind the demonstrator. A one-way door was necessary to prevent non-subject bats in the home cage from entering the maze via the exit, but opening the one-way door was not a trivial task for the exiting bats; they would sometimes push it open slightly, then give up and go back to the maze entrance. An alternative one-way exit could involve pushing through a collapsible sleeve rather than pushing a rigid door. The arrangement of the maze as tested meant that bats could learn the exit's location from demonstrators but probably learned the flap door mechanism on their own. Naive bats could go where they had seen or heard the demonstrator going, but could not observe the demonstrator opening the flap unless they were immediately behind it. The probability of documenting social learning might therefore be greater if naive bats could observe the exit task of pushing open the flap door from any point in the maze. A future experimental design should require learning of a spatial route or an instrumental task, but not both simultaneously.

Another potential constraint of our testing paradigm was that the demonstrators might have been less motivated to leave the maze in test trials because their mother and another female groupmate remained in the maze. The presence of other bats in the maze might have reduced the social reward of exiting. Indeed, in one trial, the demonstrator appeared to be about to exit, but then returned to the other bats inside the maze. To prevent this situation, the same procedure could be applied to triads of one observer and two paired demonstrators, with each demonstrator trained to exit the maze via a different exit, rather than testing two observers and one demonstrator trained to exit the maze from one exit. In this case, a naive bat that did not follow or copy either demonstrator would be alone.

The naive exit rate during demonstrator trials was lower than we had anticipated. The low sample of social learning events prevented one of our original goals: to test whether social learning was more common between mother–offspring pairs. The few learning events we did observe did not suggest a strong effect of kinship: only two of the five cases of social learning involved mother–offspring pairs, while the remaining three involved non-kin. The problem of a low exit rate could be ameliorated by repeating this experimental procedure using the same or different maze configurations to create more opportunities for social learning.

Although the evidence for social learning we report here is weak, the results of this pilot experiment are most consistent with the hypothesis that some of the naive bats learned socially, but not by following the demonstrators (social learning without following). Asocial learning is unlikely because naive exit rates decreased after the first training session (with no demonstrators), but increased after the first test trial (trained demonstrators added); i.e. the addition of demonstrators increased the probability of naive bats to successfully exit. The naive bats did not closely follow the demonstrators out of the maze, nor did they learn by observing how the trained bats opened the exit flap. In three of the five cases, the naive bats entered the exit arm after the demonstrator, but they actually exited the maze before the trained demonstrator. If social learning did occur, the most likely mechanism is that the trained demonstrators attracted the naive bats' attention to the exit arm of the maze (local or stimulus enhancement [13,16]), which then led some of the naive bats to learn the exit route.

Our general experimental paradigm represents a promising approach for testing the possible factors that affect social learning in vampire bats. Each trial required only 15 min and maze difficulty could be manipulated to achieve different learning rates in future studies. Our results suggest that social rewards can be a fast and effective alternative method for training vampire bats, compared with food rewards, which require fasting [17,18], or punishments such as electric shocks, which require a custom-built apparatus and are perhaps less ecologically relevant [19]. Given their reliance on cooperative relationships, we suggest that vampire bats might be a promising non-primate model for testing the interactions between cooperative relationships and use of social information.

## Supplementary Material

Exit data for all training sessions and test trials

## Supplementary Material

Code for data analysis

## Data accessibility

Data and R code have been uploaded as the electronic supplementary material.
